# Nickel Chalcogenide Nanoparticles-Assisted Photothermal Solar Driven Membrane Distillation (PSDMD)

**DOI:** 10.3390/membranes13020195

**Published:** 2023-02-04

**Authors:** Donia Elmaghraoui, Imen Ben Amara, Sihem Jaziri

**Affiliations:** 1 Laboratoire de Physique de la Matiére Condensée, Faculté des Sciences de Tunis, Campus Universitaire, El Manar 2092, Tunisia; 2 Laboratoire de Physique des Matériaux, Structures et Propriétés, Faculté des Sciences de Bizerte, Jarzouna 7021, Tunisia

**Keywords:** photothermal, nanoparticles, nickel chalcogenides, membrane distillation

## Abstract

Developing photothermal solar driven membrane distillation (PSDMD) is of great importance in providing fresh water for remote off-grid regions. The production of freshwater through the PSDMD is driven by the temperature difference between feed and distillate sides created via the addition of efficient photothermal nanostructures. Here we proposed nickel sulfides and nickel tellurium nanoparticles (NPs) to be loaded into the polymeric membrane to enhance its performance. Ag and CuSe NPs are also considered for comparison as they are previously used for membrane distillation (MD). Our theoretical approach showed that all of the considered NPs increased the temperature of the PVDF membrane by around a few degrees. NiS and ${\mathrm{NiTe}}_{2}$ NPs are the most efficient solar light-to-heat converters compared to NiTe and ${\mathrm{NiS}}_{2}$ NPs due to their efficient absorption over the visible range. PVDF membrane loaded with 25% of NiCs NPs and a porosity of 32% produced a transmembrane vapor flux between 22 and 27 L/m^2^h under a 10-times-amplified sun intensity. Under the same conditions, the PVDF membrane loaded with CuSe and Ag NPs produced 15 and 18 L/m^2^h of vapor flux, respectively. The implantation of NPs through the membrane not only increased its surface temperature but also possessed a high porosity which provided a higher distillation and energy efficiency that reached 58% with NiS NPs. Finally, great agreement between our theoretical model and experimental measurement is obtained.

## 1. Introduction

Nowadays the world is suffering from water scarcity due to global warming, pollution and water over usage. To overcome this issue, there has been an increasing demand for purifying abundant alternative sources such as seawater or high salinity processed water [1,2,3]. Among purifying technologies, osmosis inverse (RO) [4,5] and thermal desalination [6,7] are considered as promising technologies that turn seawater into freshwater, but at a high cost and with high energy consumption [8]. Recently, membrane distillation (MD), an hybrid membrane thermal system, has been introduced as a sustainable solution for seawater desalination [9,10,11] that distills water at lower temperatures and pressures [12,13]. Despite convincing results and significant importance, the MD efficiency is severely reduced because of the temperature polarization which consists in the reduction of membrane temperature compared to the bulk feed water [14,15,16]. This decreases the cross-membrane vapor pressure difference, hence the membrane flux. However, it has been demonstrated [16,17,18] that heating the feed water at the feed–membrane interface instead of heating the bulk feed stream outside the membrane module can reverse the temperature polarization with an enhancement of the evaporation process. Thus, the heating of the membrane surface can be obtained by the addition of photothermal materials such as plasmonic [16,17,19] or carbonic materials [18,20] to the MD system. In fact, a quite high light-to-heat conversion efficiency was achieved with these materials, leading to the increasing membrane temperature. The addition of photothermal nanostructures not only reverses the membrane temperature polarization, but also opens the way for the development of a new generation of membrane distillation powered directly by solar energy [18,21,22,23,24,25,26,27,28,29,30]. These solar driven membrane distillation (SDMD) systems are designed with physically separated solar thermal collectors and membrane distillation modules [31,32]. Nevertheless, the proposed prototype is difficult to integrate into isolated seats because of its complexity and its high production costs. Recent works have proposed an integrated solar membrane distillation prototype with the membrane distillation modules built directly into the evacuated solar tubes. This was possible via the integration of films containing photothermal nanostructures onto the membrane [18,22]. The role of these nanostructures with highly photothermal heating, induced via solar illumination, is to drive the distillation process without the requirement of heating the input water. Another study has considered a direct contact between the membrane and photothermal nanostructures (SiO2/Au nanoshell, CB NPs, and Fe_3_O_4_ NPs) [33,34,35]. The NPs are coated at the membrane surface and demonstrate that thermal energy is transmitted rapidly from the nanoparticles to the polymeric membrane via direct contact. On the other hand, the increasing concentration of coating nanoparticles, which enhances heat production, ends with reducing the vapor flux; many of the base membrane’s pores were blocked by the NPs, which resulted in a decrease in vapor permeability [35]. The best solution is to implant photothermal nanostructures through the polymeric membrane. This strategy will not only simplify the membrane fabrication process but may also improve the membrane hydrophilicity and enlarge the pores in the membrane [36]. Structural investigations demonstrated that NPs, entrapped in the PVDF membrane during the demixing process, were well dispersed throughout the polymeric matrix [16]. However, many implanted NPs either involve expensive composites or absorb solar energy in a limited spectral range. The challenge remains to introduce materials with low cost, low toxicity and proficiency in harvesting the full range of the solar spectrum.

Transition metal chalcogenide (TMCs) NPs have been proposed for use in photothermal therapy (PTT) treatments for cancer [37,38] and antibacterial membranes [39]. More recently, S. Abramovich et al. [40] have shown that embedding NiSe and CoSe nanofillers into the polymeric membrane increases the transmembrane flux by 330% and 690%, respectively. These materials have strong photo absorption characteristics in a wide range of wavelengths and have shown to be less toxic than graphene, suggesting they might have a wide range of water treatment and biomedical applications. TMC nanostructures can also degrade toxic environmental contaminants into nonhazardous products [39,41,42]. An efficient photothermal candidate must exhibit a metal-like character and show like localized plasmonic resonances absorption in particular spectral regions. Depending on their composite elements, TMCs can exist as semiconductors, semi-metals, or topological insulators [39,40,41,42,43,44]. TMC semi-conductors show metal-like absorption but typically operate in the deep UV. Among a variety of semi-metal or topological insulator TMCs, nickel chalcogenide (NiCs) series (Se, S, Te) demonstrate high metallic features due to the high degree of covalency in the nickel–chalcogen bond [43,44]. Further, NiCs are also demonstrated with a high chemical stability and ease of synthesis [37,38,45].

In this regard, the aim of this theoretical study is to push NiC NPs into the pool of desalination to invite more research investigations. Here we propose an innovative PSDMD system made by poly (vinylidenefluoride) (PVDF) materials loaded with nickel sulfides and nickel tellurides nanoparticles (NPs). The manuscript is structured as follows. In section II, we introduce the theoretical approach used to determine the membrane temperature profile and the resulting transmembrane vapor flux. In section III, we firstly discuss the absorption cross section of several types of photothermal NPs to define the most efficient absorber for the solar spectrum. Then we compare our calculation to the experimental measurement for validation. After, we illustrate the profile of membrane temperature under different intensities of solar radiation. We also compare the amount of vapor flux produced by different loaded PVDF membranes. Finally, we treat the impact of increasing the porosity of composite membrane on the performance of PSDMD.

## 2. Materials and Methods

**Figure 1:**
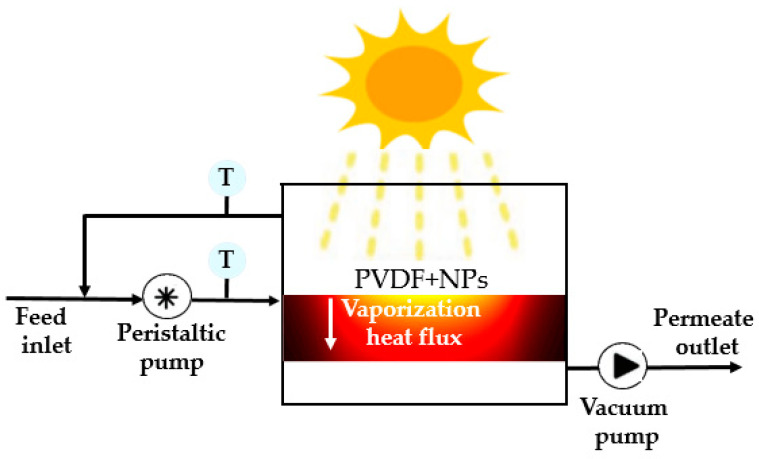
Schematic design of the solar driven membraneprototype. The NPs increase the temperature of the membrane at its surface thanks to their efficient light-to-heat conversion. The difference in temperature between the two sides of the membrane creates a gradient of vapor concentration. This helps the diffusion of vapor flux through the PVDF membrane and enhances its rate.

The schematic design of the PSDMD proposed in this theoretical study is depicted in Figure 1. This system uses a porous hydrophobic membrane that separates a hot feed from a cold distillate. The partial vapor pressure difference between each side of the membrane drives membrane vapor flux, generating pure water upon condensation. The membrane materials are inert hydrophobic polymers including poly (vinylidenefluoride) (PVDF) loaded with NiC NPs.

The experimental realization of the nanocomposite films, i.e., polymer membrane loaded with NiC NPs, was presented by P. Riddy et al. [46]. Thus, the nanocomposite films were fabricated using a cost-effective solution casting technique by dispersing different contents of NiC NPs in the polymeric matrix. The synthesis of the NiC NPs used a green synthesis approach followed by their incorporation into the polymeric matrix [46]. These results suggest that NiC NPs were dispersed homogeneously in the polymeric matrix. Accordingly, in our theoretical approach, the implanted NPs will be supposed as uniformly distributed in the membrane geometry. The temperature rise distribution of the PVDF membrane by NiC NPs, illuminated via solar irradiation, is obtained by solving the heat flow transfer equation given using:

(1) $$\rho c\frac{\partial v\left(r,t\right)}{\partial t}=k\Delta v\left(r,t\right)+D\left(r,t\right)$$

where $\upsilon =\Delta T_{m}\left(r,t\right)$ is the membrane temperature increase, k and κ are their thermal conductivity and thermal diffusivity, respectively, ρ is the mass density, c is the specific heat and D(r,t) is the rate of generated heat density. The thermal conductivity and diffusivity are calculated using an effective medium theory as a function of the membrane porosity (ε):

(2) $$\{\begin{array}{c} k=\left(1-\varepsilon \right)k_{PVDF}+\varepsilon k_{g}\\ \kappa =\left(1-\varepsilon \right)\kappa_{PVDF}+\varepsilon \kappa_{g} \end{array}$$

where $k_{PVDF}\left(\kappa_{PVDF}\right)$ and $k_{g}$ ($\kappa_{g}$) are the thermal conductivity (thermal diffusivity) of the polymeric material and the gas phase inside the membrane pores, respectively. The rate of generated heat density will be corrected using an additional term, taking into account the loss of heat via conduction through the membrane wall, defined as [17]:

(3) $$D\left(r,t\right)=\frac{ℒ\left(z\right)}{k}-\frac{\upsilon \left(r,t\right)}{\kappa \tau }$$

where τ is a cooling time constant included to describe the loss of heat inside the PVDF membrane, whatever its form. This term will be deduced from experimental measurement [16]. ℒ is regarded as effectively continuous heat production, due to the absorbed energy on NPs and is described by the exponential decrease across the thickness of the PVDF membrane:

(4) $$ℒ\left(z\right)=N_{pi}\sigma_{abs}\left(\lambda \right)Ie^{\alpha_{c}\left(z-z_{0}\right)}$$

where $N_{pi}$ is the number of illuminated particles per unit of volume, $\alpha_{c}$ is the absorption coefficient of NPs embedded in the PVDF membrane ($\alpha_{c}=N_{p}\sigma_{abs}$) and I is the solar irradiation intensity. $\sigma_{abs}$ is the absorption cross section of individual NPs which depends on its radius and the wavelength of incident light. Regarding the size of the considered NPs (NP radius ≥ 30 nm) and their metal-like characteristics, the quantum confinement effect will be negligible and Mie theory will be sufficient for the evaluation of this parameter. More details about this approach can be found in ref [17].

To obtain an analytical solution to Equation (1), we suppose that the implanted NPs are randomly distributed in an effective plat cylinder of radius $R_{m}$ and thickness H (H $\ll R_{m}$) and that the heat source generation is thermally localized within this region. The radius $R_{m}$ is estimated using the radius of the irradiated area and H using the membrane thickness. Then, the time-dependant temperature increase in the membrane $\Delta T_{m}\left(r,z,t\right)$ inside and outside the cylindrical region is solution to the following equations:

(5) $$\{\begin{array}{c} \frac{1}{\kappa }\frac{\partial \Delta T_{m}\left(r,z,t\right)}{\partial t}=\left(\frac{\partial^{2}}{\partial r^{2}}+\frac{2}{r}\frac{\partial }{\partial r}-\frac{1}{\kappa \tau }\right)\Delta T_{m}\left(r,z,t\right)+\frac{ℒ}{k}\;\;\;\;\;\;\;\;\;\;\;\;\;\;0\leq r\leq R_{m}\\ \frac{1}{\kappa }\frac{\partial \Delta T_{m}\left(r,z,t\right)}{\partial t}=\left(\frac{\partial^{2}}{\partial r^{2}}+\frac{2}{r}\frac{\partial }{\partial r}-\frac{1}{\kappa \tau }\right)\Delta T_{m}\left(r,z,t\right)\;\;\;\;\;\;\;\;\;\;\;\;\;\;\;\;r\geq R_{m}\;\;\;\;\;\;\; \end{array}$$

Experimental measurement shows that, under continuous excitation sources, the membrane temperature tends to a stationary regime [16]. Then we could deal with the stability of the time dependent solution of equation (1) and suppose the total solution as a sum of a steady state solution $\Delta T_{ss}\left(r,z\right)\;$and a time dependent perturbation Ω(r,t): $\Delta T_{m}\left(r,z,t\right)=\Delta T_{ss}\left(r,z\right)+\Omega \left(r,t\right)$. Thus, $\Delta T_{ss}\left(r,z\right)$ demonstrates a differential equation where the solutions inside and outside the cylinder are the Bessel modified functions of the first and second kind, respectively (see supporting information). Finally, together with the boundary conditions ($\Delta T_{m}\left(r,z,t∼\infty \right)=\Delta T_{ss}\left(r,z\right)$ and $\Delta T_{m}\left(r,z,t=0\right)=0$), the time dependant temperature rise is reduced to:

(6) $$\Delta T_{m}\left(r,z,t\right)=\Delta T_{ss}\left(r,z\right)\left(1-e^{-\frac{t}{\tau }}\right)$$

The obtained solution of Equation 1 defines the temperature difference between THE feed water and membrane layer at finite r and z. It decreases while z increases, inducing a difference in temperature between the membrane’s opposite sides. This leads to a gradient of vapor concentration ∇c(T) through the PVDF membrane. The water vapor concentration at any point of the membrane is given using: $c=\frac{p_{sat}\left(T\right)}{R_{g}T}$, where $R_{g}$ is the ideal gas constant, $T\;\left(=T_{amb}+\Delta T_{m}\left(r,z,t\right)\right)$ is the membrane temperature and $p_{sat}\left(T\right)$ is the saturation vapor pressure. The gradient of concentration can be approximated using an average value at the top ($c_{\mathrm{top}}$) and the bottom ($c_{\mathrm{bottom}}$) of the membrane thickness (see supporting information). According to Fick’s first law, the vapor flux through the membrane increases due to the increase in this gradient concentration. Hence, the vapor flux will be expressed as:

(7) $$f\left(r,t\right)=-MD_{m}\nabla c\left(T\right)$$

where M is the water vapor molar mass, and $D_{m}$ is the effective diffusion coefficient of water for a tortuosity $\theta \left(=\frac{3-\varepsilon }{2}\right)$ [47] and porosity ε ($D_{m}=\frac{\varepsilon }{\theta }D_{w-air}$). Then the time-dependent transmembrane vapor flux expressed in L/m^2^ h is given using:

(8) $$F\left(t\right)=\frac{\int^{\;}f\left(r,t\right)ds}{S}$$

where *S* is the total membrane area. Another useful parameter that can be calculated to estimate the overall device efficiency is energy efficiency. Energy efficiency (EE) provides a quantitative estimation of the percentage of the heat that is effectively used to promote water evaporation through the membrane. EE is thus defined as:

(9) $$EE=\frac{FSH_{vap}}{I_{0}S_{I}}$$

where $H_{vap}$ is the evaporation enthalpy of water. $I_{0}$ and $S_{I}$ denote the sunlight intensity and the irradiated membrane area, respectively.

## 3. Results and Discussions

### 3.1. Nanoparticles Absorption Cross Section

**Figure 2:**
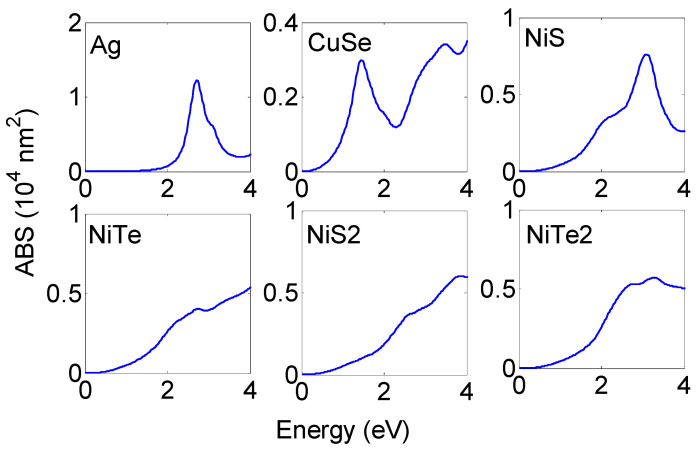
Absorption cross sections of different NP materials for 30 nm NP radius. CuSe and NiS  NPs show pronounced resonance peaks. ${\mathrm{NiTe}}_{2}$ NP resonates over the large spectral region of visible range while NiTe and ${\mathrm{NiS}}_{2}$ NPs display extended ABS in a broad spectral range from the infrared to ultraviolet.

To quantitatively characterize the absorption performance of proposed NPs, the absorption cross section (ABS) will first be discussed. Two parameters are necessary for the identification of solar light efficient absorbers: the amplitude of the absorption cross section and its energy. Figure 2 illustrates the absorption cross sections of different NP materials for a 30 nm NP radius. The absorption cross sections of Ag and CuSe NPs are also considered. CuSe and NiS NPs show pronounced resonance peaks similar to those observed with Ag NP with an amplitude of $3\times 10^{4}$ nm^2^ and $7.5\times 10^{4}$ nm^2^, respectively. These resonances are observed at 1.45 eV for CuSe NP and 3.08 eV for NiS NP. The resonance peak of ${\mathrm{NiTe}}_{2}$ NP extends over large spectral regions of visible range with an amplitude of $6\times 10^{4}$ nm^2^. NiTe and ${\mathrm{NiS}}_{2}$ NPs display extended ABS in a broad spectral range from infrared to ultraviolet. Each of the resonance peaks originated from the negative real part dielectric function in particular spectral regions; absorption resonance meets when the dielectric real part is negative and the imaginary part varies slightly with energy. As presented in Figure S1 (see supplementary materials) CuSe, NiS, and ${\mathrm{NiTe}}_{2}$ show extended deeper regions of negative$\;\varepsilon_{r}$. These regions cover the infrared spectral range for CuSe materials while being extended from infrared to visible with NiS materials and over the visible range with ${\mathrm{NiTe}}_{2}$ materials. This is not the case for NiTe and ${\mathrm{NiS}}_{2}$, where the negative region is so small and meets at a higher energy, which explained the absence of resonance peaks at a lower energy.

**Figure 3:**
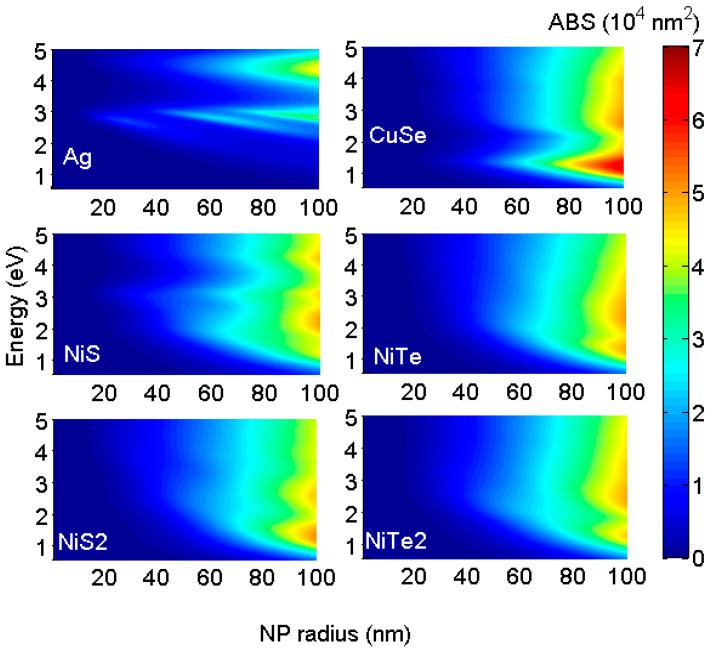
Distribution of absorption cross sections of different NPs function of energy and NP radius. All considered NPs show increasing ABS amplitude and rising new resonance peaks with increasing radius. Some of the considered NPs are good absorbers for solar light with small sizes while others need large radii for achieving resonance in the visible.

The ABS resonance peaks can be manipulated by changing some parameters. ABS resonance energy and its amplitude are sensitive to the nanostructure composition, shape, size, and surrounding medium [17]. Increasing the NP radius leads to the enhancement in ABS amplitude and the appearance of new resonance peaks. This is apparent in Figure 3, where we have plotted the ABS distribution of different NPs as a function of the NP radius. As expected, all of the considered NPs show increasing ABS amplitude and rising, new resonance peaks with an increasing radius. Some of the considered NPs are good absorbers for solar light with small sizes while others need large radii for achieving resonance in the visible. For small radii (≤30 nm), only A,g, NiS and ${\mathrm{NiTe}}_{2}$ NPs show absorption for the visible while NiTe and ${\mathrm{NiS}}_{2}$ NPs absorb over the UV. For greater sizes (beyond 30 nm), the ABS resonances of these NPs are more pronounced and cover the visible and infrared regions. Large CuSe NP shows efficient absorption for infrared (ABS $\approx 7\times 10^{4}$ nm^2^ for radius = 100 nm) and the rise of broadband resonance over the visible range (NP radius ≥ 60 nm).

In sum, NiS and${\;\mathrm{NiTe}}_{2}$ with small radii (NP radius ≤40 nm) will be excellent absorbers of solar radiation. The larger size is needed for NiTe and ${\mathrm{NiS}}_{2}$ to enhance their absorption of solar radiation. CuSe NPs are good absorbers for the infrared with resonance peaks at 1.45 eV; this is consistent with the experimental measurement [39]. This makes CuSe NPs a promising candidate for biomedical application.

### 3.2. Photothermal Membrane Temperature and Vapor Flux

**Figure 4:**
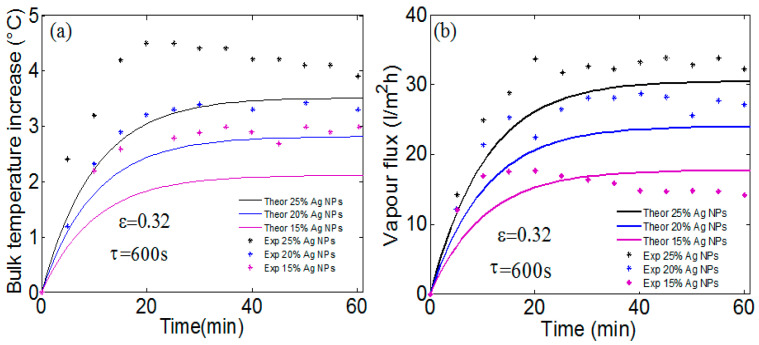
Theoretical calculations and measurement for (**a**) the membrane temperature rise and (**b**) the transmembrane vapor flux to pure water. The cooling time is obtained by fitting the measured bulk temperature increase using an exponential function. PVDF membranes loaded with 25% Ag NPs can produce vapor flux around 11-fold higher than the corresponding values for unloaded membranes.

The absorption of different NPs determines the heat source density which dictates the solar photothermal temperature rise and thus the water vapor flux. As discussed in previous works [16,22], the membrane-localized heating via illuminated NPs dominates the total produced heat and induces vaporization of the feed water. This will be confirmed by our simulation for experimental measurements obtained by A. Politano et al. [16] on PVDF membrane loaded with Ag NPs. Figure 4a,b show a comparison between our theoretical calculations and the experimental measurement [16] for the membrane temperature rise and the transmembrane vapor flux to pure water, respectively. The experimental measurement was obtained using a high-pressure UV mercury lamp, with a wavelength of 366 nm, and a viewing angle of 90° is used to irradiate the membrane area of 21.24 cm^2^ [16]. Three different compositions of Ag NPs are considered: magenta, blue and black curves indicate a 15%, 20% and 25% of Ag NPs composition in the membrane, respectively. The incident light is about 23 kW/m^2^, respectively. The cooling time τ is equal to 600 s and is obtained by fitting the measured bulk temperature increase using an exponential function: $\Delta T\left(t\right)=A\left(1-\exp\left(-\frac{t}{\tau }\right)\right)$.

The temperature rise is calculated at the center of the membrane surface (r=0) for an ambient temperature of 21 °C and a porosity of 32%. Great agreement between calculation and measurement for both the temperature rise and transmembrane vapor flux is obtained. The small difference is due to the choice of cooling time, where a greater value is possible and may lead to increasing membrane absorption and a temperature rise. In addition, the contribution of UV lamp heat to the total produced heat has not been considered. One can note that the PVDF membranes loaded with 25% Ag NPs can produce vapor flux by about 30 L/m^2^ h, which is 10-fold higher than the corresponding values for unloaded membranes [16] Despite their efficient photothermal conversion under UV radiation, Ag NPs will not be of great interest to solar sources where broadband absorption over the visible range is needed.

**Figure 5:**
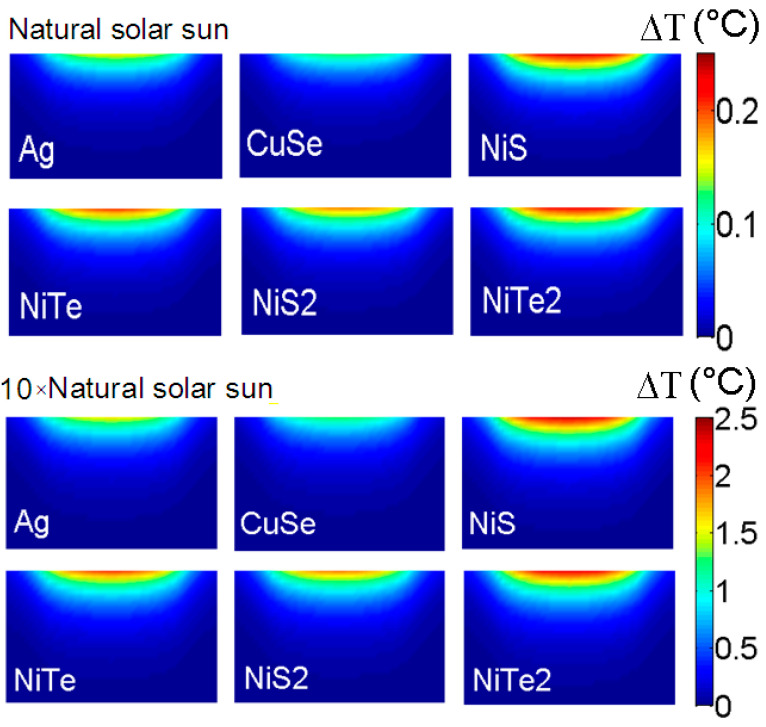
The temperature rise profile of PVDF membrane under solar radiation (λ=400−800 nm) with two different intensities. The temperature rise of PVDF membrane loaded with NiS and NiTe2 NPs reaches 0.25 °C and 2.5 °C (at the membrane surface center) under natural solar sun radiation and its amplified intensity, respectively.

The accuracy between our theoretical model and the experimental measurement allows us to theoretically examine the membrane temperature rise as a function of implanted NPs. As discussed before, NiS and ${\mathrm{NiTe}}_{2}$ show large important absorption over the visible range, which can enhance the membrane temperature rise under solar radiation. In addition, the heat production of NPs increases with increasing excitation intensity due to their linear dependence. This is shown in Figure 5, where we present the temperature rise profile of the PVDF membrane under solar radiation (λ=400−800 nm) with two different intensities. The solar spectral irradiance is considered at sea level (see supporting information). The PVDF membrane is loaded with 25% of different NPs (including Ag NPs) with a radius of 40 nm. We can see an increase of about 10 times in the PVDF temperature rise while the intensity is amplified. The temperature rise of the PVDF membrane loaded with NiS and ${\mathrm{NiTe}}_{2}$ NPs reaches 0.25 °C and 2.5 °C (at the membrane surface center) under natural solar sun radiation and its amplified intensity, respectively. A less-heated membrane surface is obtained with NiTe and ${\mathrm{NiS}}_{2}$ NPs, where an increase of ~2 °C is observed with amplified solar radiation.

Due to their lower absorption for visible (compared to the NiCs NPs), CuSe and Ag NPs produce lower heat under solar radiation where a rise of ~1 °C is obtained for amplified intensity. In spite of this, the implantation of CuSe NPs through the membrane can stop the penetration of contaminants and hence the deterioration of treated water quality [39]. It is worth noting that for different loaded membranes, the temperature rise is about 0 °C at the bottom of the membrane (distillate side). Hence, the difference in temperature between the two sides will be manipulated by the nature of implanted NPs.

### 3.3. Membrane Efficiency

The evaluation of membrane efficiency is the key parameter for the technical realization of PSDMD. In a membrane system the contact angle, pore size and vapor flux are critical. However, the implantation of NPs into the polymeric membrane not only increases its temperature and vapor flux but also increases its pore size and contact angle (>110°). As a result, the membrane exhibited much higher resistance to wetting [16,40]. In this regard, the wetting effect will be neglected and only the effects of implanted NPs and porosity on the membrane efficiency will be discussed.

**Figure 6:**
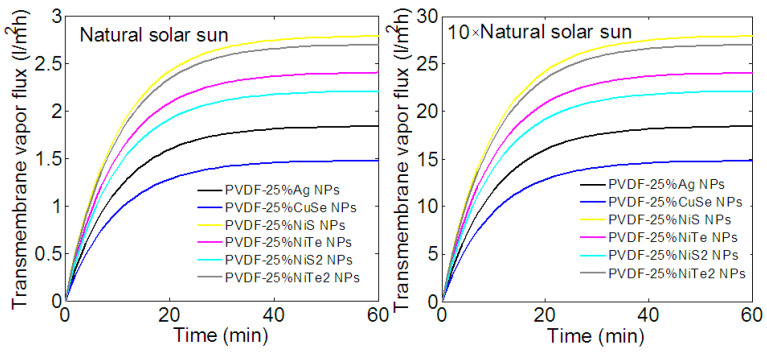
Time-dependent transmembrane vapor flux of different composite membranes for two solar intensities. After 1 h of illumination the highest transmembrane vapor flux is about 26–27 L/m^2^h for amplified intensity and 2.7 L/m^2^h under the natural sun. A membrane loaded with NiTe and ${\mathrm{NiS}}_{2}$ NPs can produce about 22 and 24 L/m^2^h of vapor flux.

Let us first discuss the effect of implanted NPs on the production of vapor flux. As expected, the transmembrane vapor flux increases with the amplification of solar irradiation intensity and the nature of injected NPs. As shown in Figure 6, after one hour of illumination, the highest transmembrane vapor flux is about 26–27 L/m^2^h for amplified intensity and ~2.7 L/m^2^h under the natural sun. These values, which are obtained with NiS and ${\mathrm{NiTe}}_{2}$ NPs, are approximately half more than those obtained with Ag NPs. A lower vapor flux is obtained when the membrane is loaded with 25% of NiTe and ${\mathrm{NiS}}_{2}$ NPs; it can produce about 22 and 24 L/m^2^h of vapor flux under an amplified intensity. Despite their weak photothermal effect under solar radiation, the implantation of CuSe NPs helps to produce 15 L/m^2^h of vapor flux.

The increasing transmembrane vapor flux with temperature can be explained if we consider how the vapor concentration (through the membrane) depends on temperature. In a composite photothermal membrane, the temperature is maximal at the feed PVDF membrane interface and decreases along its thickness (see Figure 5). Hence, the feed flow increases its temperature close to the PVDF membrane and its water vapor concentration. The difference in temperature between the hot and cold sides produced a higher flux because of the gradient of vapor concentration along the membrane thickness. With increasing intensity and efficient photothermal NPs, this temperature difference increases, leading to an increasing vapor flux. We can note that the excellent water vapor generation performance of the PVDF-25% NiC NPs composite membrane is associated with the high light absorption range of the NiC NPs over the visible solar spectrum and good photothermal conversion efficiency.

**Figure 7:**
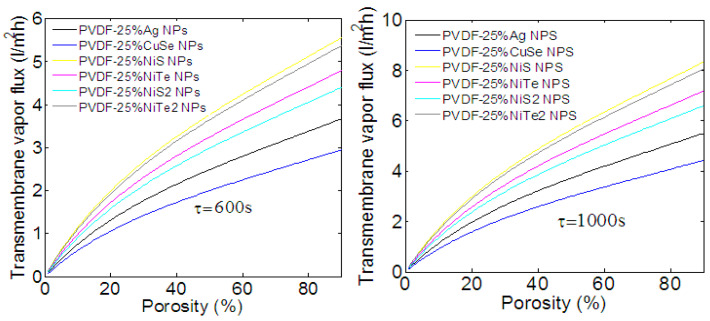
Transmembrane vapor flux as a function of the porosity. PVDF membrane loaded with different TMCs NPs produces between 3 and 5.5 L/m^2^h of vapor flux. These values increase to 4.2 and 8 L/m^2^h when the cooling time is about 1000 s, i.e., lower loss of heat.

All of the physical parameters (porosity and cooling time) used in the latter simulation are taken from ref. [16], where the PVDF membrane is loaded with Ag NPs. However, the nature of NPs and their sizes affect the porosity, which changes the membrane’s thermal conductivity and diffusivity. In fact, the implantation of NPs through the membrane increases the size of its pores, leading to an increasing porosity and a decreasing loss of heat, i.e., long cooling time. Analyzing these effects through the PVDF membrane is important to enhance the performance of the nickel chalcogenide composite PVDF membrane. We further investigate the transmembrane vapor flux and the energy efficiency under the variation of porosity and cooling time. As discussed in previous experimental measurements, the porosity varies from 27 to 56% with the nature and composition of injected NPs [16,40]. Other works show that the porosity of composite membranes is still as high as 85% [22]. According to these results, the porosity will be varied from 0 to 90%. The variation of transmembrane vapor flux as a function of the porosity and for two cooling times is depicted in Figure 7. As we can see, under solar radiation, a cooling time of 600 s, and the maximum of porosity (about 90%), a PVDF membrane loaded with different NPs produces between 3 and 5.5 L/m^2^h of vapor flux. This corresponds, as calculated in Equation (9) and indicated in Figure S2 (see supplementary materials), to the energy efficiency of 28 and 52% (these values increase to 4.2 and 8 L/m^2^h when the cooling time is about 1000 s, supposedly 41 and 80% of energy efficiency. Figure S2 also shows that, for 32% of porosity and a cooling time of 600 s, which corresponds to a PVDF membrane loaded with Ag NPs [16], the energy efficiency is about 26% for the PVDF membrane loaded with 25% of NiS NPs. However, a porosity of about 56% is obtained when the membrane is loaded with NiCs NPs [40], and a higher energy efficiency and long cooling time is thus expected. Then, for a porosity of 56% and a cooling time of 1000 s, Figure S2 shows an energy efficiency of 58% for a membrane loaded with NiS NPs. This result is in good agreement with recent experimental measurements [40].

The increasing energy efficiency with increasing porosity can be explained via the higher diffusion coefficient and the lower loss of heat. The higher diffusion coefficient helps the diffusion of vapor flux through the membrane. The lower loss of heat, which is translated by a long cooling time, leads to an increasing temperature at the membrane surface. Hence, the difference in temperature between opposite sides of the membrane increases, resulting in an increased vapor flux. We can conclude that the composite membrane possesses a high porosity, which provides a higher distillation and energy efficiency. All of the considered NPs show efficient photothermal conversion in a particular spectral region. However, as the variation of porosity as a function of most of the implanted NPs is not defined, we are not able to define exactly the most efficient photothermal candidate, and thus more experimental investigations are needed.

## 4. Conclusions

To summarize, we proposed transition metal chalcogenide NPs to be loaded to the hydrophobic membrane to enhance the performance of the solar-driven membrane. Our theoretical investigation has demonstrated that nickel chalcogenide (S and Te) nanoparticles were the most efficient photothermal absorbers of solar radiation. CuSe and Ag NPs showed a lower absorption for solar radiation, and are even more effective in the infrared and ultraviolet regions, respectively. All of the considered NPs raised the temperature of the membrane surface compared to the feed temperature. The increasing difference in temperature between the opposite sides of the membrane has driven the production of vapor flux through the membrane, which reached 27 L/m^2^h with NiS NPs and obtained a smaller heat input. In addition to their photothermal capacities, the implantation of nickel chalcogenide NPs through the PVDF membrane increased its pore size and wetting resistance, leading to a higher energy efficiency. Accordingly, our investigation showed an energy efficiency of 58% for a PVDF loaded with NiS NPs, which is in good agreement with a recent experiment [40]. Finally, we hope that our proposed approach helps other experiments to develop a new generation of photothermal membrane distillation and encourage the use of nickel chalcogenide NPs for such applications. Thanks to their efficient absorption for solar spectra, these NPs can be also used to enhance the absorption of thin film solar cells.

## Data Availability

The data supporting reported results are available from the corresponding author upon reasonable request.

## Supplementary Materials

The following supporting information can be downloaded at: https://www.mdpi.com/article/10.3390/membranes13020195/s1, Figure S1: dielectric function of all of considered materials; Figure S2: energy efficiency of different composite membranes at different porosity and cooling times; Figure S3: solar spectral irradiance at sea level; Table S1: different parameters used to calculate the membrane temperature and vapor flux [47,48,49,50,51,52,53,54].
