# Supplementary material for: Nickel Chalcogenide Nanoparticles-Assisted Photothermal Solar Driven Membrane Distillation (PSDMD)
Source: Membranes (Basel). 2023 Feb 4;13(2):195. doi: 10.3390/membranes13020195 (PMC9961219; doi:10.3390/membranes13020195)
Supplement: Supplementary file 1 [file membranes-13-00195-s001.zip › membranes-1961291-supplementary.pdf]

SUPPORTING INFORMATION

Nickel Chalcogenide nanoparticles-assisted Photothermal Solar Driven Membrane Distillation (PSDMD)

Donia Elmaghraoui, Imen Ben Amara, and Sihem Jaziri

1.Dielectric function

Light to heat conversion in metallic NPs is governed by plasmonic resonances. However, it has shown that similar optical resonance can be achieved in other nanostructures made of semi-metal or topological insulators from the p-block; they behave optically as a metal in distinguish spectral region. Metal like optical resonance is met when the values of the material complex dielectric function ($\boldsymbol{\varepsilon} = \boldsymbol{\varepsilon_r} + \boldsymbol{i\varepsilon_i}$) show a negative real part ($\boldsymbol{\varepsilon_r} < \mathbf{0}$) and a small enough imaginary part $\boldsymbol{\varepsilon_i}$. Therefore, the dielectric function will be the key parameter for the identification metal like materials. Here we interest to Nickel Chalcogenides series (S, Te). In particular, we will consider the dielectric function of NiS, NiTe, $\mathbf{NiS_2}$, $\mathbf{NiTe_2}$ and CuSe. The dielectric function $\boldsymbol{\varepsilon(\omega)}$, which describes the response of the semi-metals (NiS, NiTe and CuSe) and topological insulators ($\mathbf{NiS_2}$ and $\mathbf{NiTe_2}$) to external excitations, is elucidated within the density functional theory (DFT) [1] using the full potential linearized augmented plane wave method plus local orbital method (FP-LAPW+lo) [2] as implemented in Wien2k code [3]. The (FP-LAPW+lo) approach consists on solving the Kohn–Sham equations [4]. Accordingly, the imaginary part of $\boldsymbol{\varepsilon(\omega)}$ is given in the momentum representation in terms of indirect and direct interband transitions, where the transitions satisfying the dipole selection rule Δl= ±1 are considered.

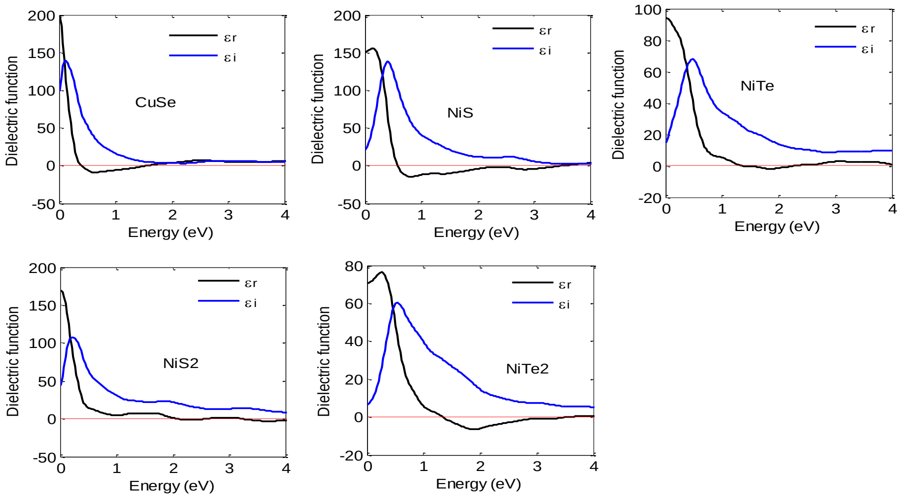

Figure S1: The dielectric function $\boldsymbol{\varepsilon} = \boldsymbol{\varepsilon_r} + \boldsymbol{i\varepsilon_i}$ of bulk CuSe, NiS, NiTe, NiS2 and NiTe2. All of considered materials show an anomalous jump of εr spectrum from positive to negative values while the spectrum of εi show peaks located at the far-mid infrared.

However, the indirect ones involve the scattering of phonon are neglected due to the corresponding small contribution. Thus, $\boldsymbol{\varepsilon(\omega)}$ can be obtained using the joint total density of states (DOS) of the present materials and the optical matrix elements $\boldsymbol{M_{cv}(k)}$ [2] as follows:

$$\varepsilon(\omega)=\frac{8\Pi^2e^2}{\Omega m^2\omega^2}\sum_{c}^{unocc}\sum_{v}^{occ}\int BZ\ \ |M_{cv}(k)|^2 f_{vk}(1-f_{ck})\delta\,(E_{ck}-E_{vk}-\hbar\omega)$$

In this notation, m is the electron mass, Ω is the unit cell volume and $f_{c(v)k}$ is The Fermi distribution function. The term $\delta\,(E_{ck}-E_{vk}-\hbar\omega)$ assures the total energy conservation. Here, we note that the integral is over the first Brillouin zone (BZ). Consequently, the real part dielectric constant can be derived via Kramers–Kronig transformation [5]. Figure S1 shows the dielectric function real and imaginary parts for different Bulk materials. All of considered materials show an anomalous jump of ε_r. spectrum from positive to negative values while the spectrum of ε_i.show peaks located at the far-mid infrared. The contributions of free carriers and interband transition to ε are at the origin of these features. The existence of peaks on ε_i spectrum is attributed to the interband transitions while the jump of ε_r at lower energy (far-mid infrared) is due to the contribution of free carriers. The resonance of ε_r at lower energy is a signature of a high oscillator strength of interband transitions.

From this conception, we can deduce that the dielectric function of CuSe is dominated by the free carrier distribution which induces negative region of ε_r in the infrared. For NiS the jump of ε_r at lower energy (far-Infrared) is due to important contribution of free carriers while its extension over the visible is attributed to interband transitions with important oscillator strength. NiTe and $\mathbf{NiS_2}$ show approximately similar characteristics where the jump is located at higher energy (edge of infrared and visible) indicating the negligence of free carriers contribution and the dominance of interband transition with lower oscillator strength. This is not the case for $\mathbf{NiTe_2}$ where the contribution of large interband transitions with high oscillator strength is at the origin of the deep extension of negative ε_r over the visible range.

2.Time dependent membrane temperature increase:

The temperature rise distribution of homogenous medium by a set of photothermal NPs is obtained by solving the heat flow transfer equation given by:

$$\rho c\frac{\partial v(r,t)}{\partial t}=k\Delta v(r,t)+D(r,t) \tag{S1}$$

where $v=\Delta T_m(r,t)$ is the medium temperature increase, k and $\kappa=k/\rho c$ are the thermal conductivity and thermal diffusivity of the medium, respectively, ρ is the mass density, c is the specific heat and $D(r,t)$ is the rate of generated heat density. $D(r,t)$ is corrected by an additional term taking into account the loss of heat through the membrane wall and defined as : $D(r,t)=\frac{\mathcal{L}(z)}{k}-\frac{v(r,t)}{\kappa\tau}$. Here $\mathcal{L}$ is the continuous heat production, due to absorbed energy on NPs and is described by exponential decrease across the thickness of PVDF membrane: $\mathcal{L}(z)=N_{pi}\sigma_{abs}(\lambda)Ie^{\alpha_c z}$. Where N_{pi} is the number of illuminated particles per unit of volume, α_c is the absorption coefficient of NPs embedded in the PVDF membrane ($\alpha_c=N_p\sigma_{abs}$) and I is the solar irradiation intensity. σ_{abs} is the absorption cross section of individual NP which depends on its radius and the wavelength λ of incident light.

In order to obtain an analytical solution to equation (S1), we suppose that the implanted NPs are randomly distributed in an effective cylinder of radius R_m and thickness L (L≪ R_m) and that the heat source generation is thermally localized within this region. R_m and L are defined by the radius of irradiated area and membrane thickness, respectively. Accordingly, the time-dependent temperature increase of the medium $\Delta T_m(r,z,t)$ inside and outside the cylindrical region is reduced to:

$$\begin{cases}\frac{1}{\kappa}\frac{\partial\Delta T_m(r,z,t)}{\partial t}=\left(\frac{\partial^2}{\partial r^2}+\frac{2}{r}\frac{\partial}{\partial r}-\frac{1}{\kappa\tau}\right)\Delta T_m(r,z,t)+\frac{\mathcal{L}}{k} & 0\le r\le R_m\\ \frac{1}{\kappa}\frac{\partial\Delta T_m(r,z,t)}{\partial t}=\left(\frac{\partial^2}{\partial r^2}+\frac{2}{r}\frac{\partial}{\partial r}-\frac{1}{\kappa\tau}\right)\Delta T_m(r,z,t) & r\ge R_m\end{cases} \tag{S2}$$

The experimental results show that the membrane temperature tends to a stationary regime under continuous excitations. Then we could deal with the stability of the time dependent solution of equation S2 and suppose that the total solution is a sum of a steady-state solution and a time dependent perturbation: $\Delta \boldsymbol{T_m}(\boldsymbol{r},\boldsymbol{z},\boldsymbol{t}) = \boldsymbol{\Delta T_{ss}}(\boldsymbol{r},\boldsymbol{z}) + \boldsymbol{\Omega}(\boldsymbol{r},\boldsymbol{t})$. The steady-state solution is obtained by:

$$\left(\frac{\partial^2}{\partial r^2} + \frac{2}{r}\frac{\partial}{\partial r} - \frac{1}{\kappa\tau}\right)\Delta \boldsymbol{T_{ss}}(\boldsymbol{r},\boldsymbol{z}) + \frac{\mathcal{L}(z)}{k} = \boldsymbol{0} \qquad \text{(S3)}$$

Then $\Delta \boldsymbol{T_{ss}}\ (\boldsymbol{r},\boldsymbol{z})$ is given by:

$$\begin{cases} \Delta \boldsymbol{T_{ss}}(\boldsymbol{r},\boldsymbol{z}) = \boldsymbol{A I_0}(\boldsymbol{\alpha r}) + \frac{\mathcal{L}(z)\kappa\tau}{k} & \boldsymbol{0} \le \boldsymbol{r} \le \boldsymbol{R_m} \\ \Delta \boldsymbol{T_{ss}}(\boldsymbol{r},\boldsymbol{z}) = \boldsymbol{C K_0}(\boldsymbol{\alpha r}) & \boldsymbol{r} \ge \boldsymbol{R_m} \end{cases} \qquad \text{(S4)}$$

Where $\boldsymbol{\alpha} = (\boldsymbol{\kappa\tau})^{-1/2}$, $\boldsymbol{I_0}$ and $\boldsymbol{K_0}$ are the Bessel-modified functions of the first and second kind respectively. A and B are chosen to guarantee the continuity of solutions and their first derivative at $\boldsymbol{r} = \boldsymbol{R_m}$. Then the time dependant perturbation is a solution for the following equation:

$$\frac{1}{\kappa}\frac{\partial\Omega(r,t)}{\partial t} = \left(\frac{\partial^2}{\partial r^2} + \frac{2}{r}\frac{\partial}{\partial r} - \frac{1}{\kappa\tau}\right)\boldsymbol{\Omega}(\boldsymbol{r},\boldsymbol{t}) \qquad \text{(S5)}$$

Since this equation is linear we may introduce an exponential time dependence in $\boldsymbol{\Omega}$ so that $\boldsymbol{\Omega}(\boldsymbol{r},\boldsymbol{t}) = \boldsymbol{s}(\boldsymbol{r})\boldsymbol{e^{\sigma t}}$. Then equation S5 yields:

$$\frac{\sigma}{\kappa}\boldsymbol{s}(\boldsymbol{r}) = \left(\frac{\partial^2}{\partial r^2} + \frac{2}{r}\frac{\partial}{\partial r} - \frac{1}{\kappa\tau}\right)\boldsymbol{s}(\boldsymbol{r}) \qquad \text{(S6)}$$

We obtain $\boldsymbol{\sigma} = -\frac{\mathbf{1}}{\tau}$. Finally, with the initial condition ($\Delta \boldsymbol{T_m}(\boldsymbol{r},\boldsymbol{z},\boldsymbol{t} = \boldsymbol{0}) = \boldsymbol{0}$) the time dependent temperature rise is reduced to:

$$\Delta \boldsymbol{T_m}(\boldsymbol{r},\boldsymbol{z},\boldsymbol{t}) = \Delta \boldsymbol{T_{ss}}(\boldsymbol{r},\boldsymbol{z})(\boldsymbol{1} - \boldsymbol{e}^{-\frac{t}{\tau}}) \qquad \text{(S7)}$$

3.Transmembrane vapor flux

The dependent time rate of water expressed in liter/hour is given by:

$$\boldsymbol{J}(\boldsymbol{t}) = \int \boldsymbol{f ds} \qquad \text{(S8)}$$

Where $\boldsymbol{f}$ is the vapor flux and increases due to an increasing gradient of vapor concentration across the membrane thickness according to Fick's first law:

$$\boldsymbol{f} = -\boldsymbol{M D_m \nabla c} \qquad \text{(S9)}$$

Where $\boldsymbol{M}$ is the water vapor molar mass, $\boldsymbol{D_m}$ is the effective diffusion coefficient of water for a tortuosity $\boldsymbol{\theta}(= \frac{3-\varepsilon}{2})$ [6], and porosity $\boldsymbol{\varepsilon}$ ($\boldsymbol{D_m} = \frac{\varepsilon}{\theta}\boldsymbol{D_{w-air}}$), and $\boldsymbol{\nabla c}$ is the vapor concentration gradient across the membrane thickness (along the z-direction). The water vapor concentration at any point of the membrane is given by:

$$\boldsymbol{c} = \frac{p_{sat}(T)}{R_g T} \qquad \text{(S10)}$$

Where $\boldsymbol{R_g}$ is the ideal gas constant and $\boldsymbol{T}\ (= \boldsymbol{T_{amb}} + \Delta \boldsymbol{T}(\boldsymbol{r},\boldsymbol{z},\boldsymbol{t}))$ is the membrane temperature. $\boldsymbol{p_{sat}}(\boldsymbol{T})$ is the saturation vapor pressure that is approximated by an exponential equation obtained by fitting tabulated data (between 1° and 99°C) [7]:

$$\boldsymbol{p_{sat}}(\boldsymbol{T}) = \boldsymbol{p_0 e^{\gamma T}} \qquad \text{(S11)}$$

For simplicity the gradient of concentration can be approximated by an average value at the top ($\mathbf{c_{top}}$) and the bottom ($\mathbf{c_{bottom}}$) of the membrane thickness:

$$\nabla \mathbf{c} = (\frac{\mathbf{c_{bottom}} - \mathbf{c_{top}}}{\mathbf{L}})\mathbf{n} \tag{S12}$$

Where L is the membrane thickness and $\mathbf{n}$ is a unit vector orthogonal to the membrane surface. Then

$$\boldsymbol{f} = \boldsymbol{MD_m}(\frac{\mathbf{c_{top}} - \mathbf{c_{bottom}}}{\mathbf{L}})\mathbf{n} \tag{S13}$$

Finally the effective transmembrane vapor flux is expressed as:

$$\boldsymbol{F(t)} = \frac{\boldsymbol{J(t)}}{\boldsymbol{S}} = \frac{\boldsymbol{MD_m}}{\boldsymbol{LS}} \iint (\mathbf{c_{top}} - \mathbf{c_{bottom}})\boldsymbol{rdrd\theta} \tag{S14}$$

Where S is the total membrane area.

4.Energy efficiency:

Figure S3 shows the energy efficiency of different composite membranes at different porosity and cooling times. A clear increase in the energy efficiency of the different composite membrane with increasing porosity is observed.

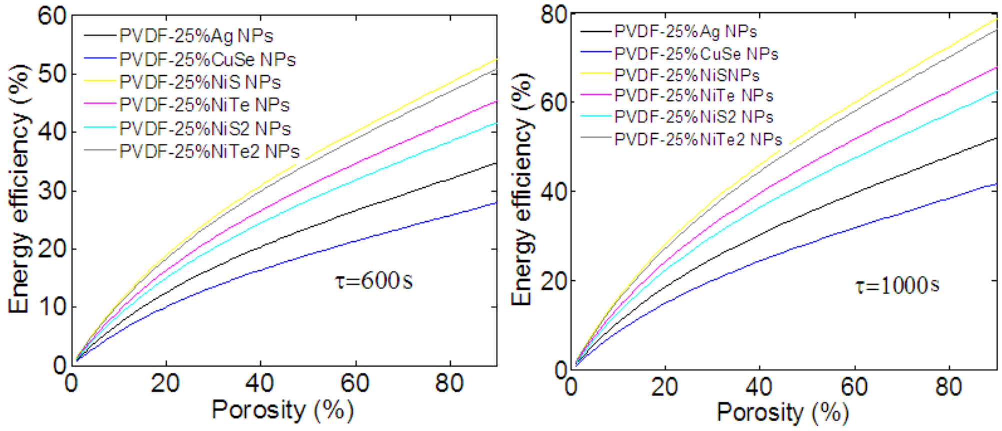

Figure S2. The energy efficiency of different composite membranes at different porosity and cooling times. PVDF membrane loaded with NiS NPs show the highest energy efficiency of 52% which reaches 80% for greater cooling time.

Under natural solar radiation (figure S3), a cooling time of 600s, and a porosity of 90%, PVDF membrane loaded with NiS NPs show the highest energy efficiency of 52%. This efficiency reaches 80% for lower loss of heat, i.e greater cooling time. For 32% of porosity, the energy efficiency is about 14, 17, 20, 23, 25 and 26 % for PVDF membrane loaded with 25% of CuSe, Ag, NiS2, NiTe, NiTe2 and NiS NPs, respectively. For each of implanted NPs the enhancement of membrane efficiency with increasing porosity is due to the higher diffusion coefficient and lower loss of heat. Hence, the amount of produced heat will be effectively used to promote water evaporation through the membrane. The enhancement of membrane efficiency with the nature of NPs is due to the increase of heat production which increases the induced vapor flux. Interestingly, we can see that for fixed excitation sources, the nature of implanted NPs and membrane porosity can be optimized to yield maximum of efficiency.

Table. S1. Different parameters used to calculate the membrane temperature and vapor flux. PVDF parameters are taken from ref [8].

$D_{w-air}(m^2/s)$	k_{PVDF} (W/mK)	$c_{PVDF}(J/Kg)$	$\rho_{PVDF}(g/cm^3)$	$k_g(W/mK)$	κ_g (mm^2/s)
2.6×10^{-6}	0.19	1.120	1.78	0.024	18.46

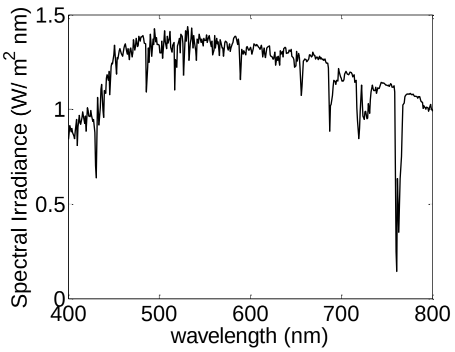

Figure S3. Solar spectral irradiance at sea level in the range 400-800 nm.

References

1. Hohenberg, P.; Kohn, W. Inhomogeneous electron gas. *Phys. Rev. B* **1964**, 136, 864.

2. Draxel, C. A.; Sofo, J. O. Linear optical properties of solids within the full-potential linearized augmented plane wave method. *Comput. Phys. Commun*. **2006**, 175, 1.

3. Schwarz, K.; Blaha, P.; Trickey, S. B. Electronic structure of solids with WIEN2k. *Mol. Phys.* **2010**, 108, 3147.

4. Kohn, W.; Sham, L. J. Self-consistent equations including exchange and correlation. *Phys. Rev*. **1965**, 140, A1133.

5. Yu. P and Cardona. M , Fundamentals of Semiconductors Physics and Materials Properties **2010**, (Berlin: Springer)

6. Shen, L.; Chen, Z.X. Critical review of the impact of tortuosity on diffusion. *Chem. Eng. Sci.* **2007**, 62, 3748-3755.

7. Lide, R. David, CRC Handbook of Chemistry and Physics **2004**, CRS Press. (85th ed), 6.

8. Buonomenna, M. G.; et al. New PVDF membranes: The effect of plasma surface modification on retention in nanofiltration of aqueous solution containing organic compounds. *Water Res* **2007**, 41:4309–4316.
